# Efficacy and safety of bendamustine for lymphodepletion before lisocabtagene maraleucel

**DOI:** 10.1186/s13045-024-01542-9

**Published:** 2024-04-22

**Authors:** Guido Ghilardi, Luca Paruzzo, Vrutti Patel, Jakub Svoboda, Emeline R. Chong, Eugenio Fardella, Elise A. Chong, Giulia Gabrielli, Sunita D. Nasta, Daniel J. Landsburg, Jordan Carter, Raymone Pajarillo, Stefan K. Barta, Griffin White, Elizabeth Weber, Ellen Napier, David L. Porter, Alfred L. Garfall, Stephen J. Schuster, Marco Ruella

**Affiliations:** 1 Lymphoma Program, Abramson Cancer Center, University of Pennsylvania, Philadelphia, PA USA; 2 Center for Cellular Immunotherapies and Cellular Therapy and Transplant, University of Pennsylvania, Philadelphia, PA USA; 3 Division of Hematology-Oncology, Hospital of the University of Pennsylvania, Philadelphia, PA USA

**Keywords:** Non-Hodgkin lymphoma (NHL), Chimeric antigen receptor T cells (CART), Lymphodepletion, Bbendamustine, Ccytokine-release syndrome (CRS), Immune effector cell associated neurotoxicity syndrome (ICANS), Toxicities, Lisocabtagene maraleucel

## Abstract

Bendamustine has been retrospectively shown to be an effective and safe lymphodepletion regimen prior to the anti-CD19 chimeric antigen receptor T cell (CART) products tisagenlecleucel and axicabtagene ciloleucel, as well as the anti-BCMA CART products idecabtagene vicleucel and ciltacabtagene autoleucel. However, bendamustine as lymphodepletion prior to lisocabtagene maraleucel (liso-cel), a 4-1BB co-stimulated, fixed CD4:CD8 ratio anti-CD19 CART product, has not been described yet. Thus, we studied a cohort of sequentially-treated patients with large B-cell lymphomas who received bendamustine lymphodepletion before liso-cel at the University of Pennsylvania between 5/2021 and 12/2023 (*n* = 31). Patients were evaluated for toxicities and responses. Of note, 7 patients (22.6%) would have dnot met the inclusion criteria for the registrational liso-cel clinical trials, mostly due to older age. Overall and complete response rates were 76.9% and 73.1%, respectively. At a median follow-up of 6.3 months, the 6-month progression-free and overall survival were 59.9% and 91.1%, respectively. Rates of cytokine-release syndrome (CRS) and neurotoxicity (ICANS) of any grade were 9.7% and 9.7%, respectively, with no grade ≥ 3 events. No infections were reported during the first 30 days following liso-cel infusion. Neutropenia ≥ grade 3 was observed in 29.0% of patients; thrombocytopenia ≥ grade 3 occurred in 9.7%. In conclusion, bendamustine lymphodepletion before liso-cel appears to be a strategy that can drive tumor responses while ensuring a mild toxicity profile.

## To the editor

Anti-CD19 chimeric antigen receptor T cell therapies (CART19) are now a standard treatment for relapsed and/or refractory large B-cell lymphoma (LBCL) patients [1]. Lymphodepletion ensures the appropriate environment for CART cell engraftment and effector functions [2]. The combination of fludarabine and cyclophosphamide (Flu/Cy) is the standard lymphodepletion regimen before commercial CART19 products, including lisocabtagene maraleucel (liso-cel) [3–5]. Bendamustine has been demonstrated to be a safe and effective lymphodepletion regimen before tisagenlecleucel [6, 7] (for which is an FDA-approved lymphodepletion ) and, more recently, axicabtagene ciloleucel [8, 9]. However, data regarding the efficacy of bendamustine before liso-cel, a 4-1BB co-stimulated, fixed CD4:CD8 T-cell ratio product, are lacking.

To this goal, we retrospectively evaluated 31 LBCL patients treated with bendamustine lymphodepletion (90 mg/m^2^ daily over two days) followed by liso-cel at the University of Pennsylvania between 5/2021 and 12/2023 (Table 1). The data cut-off date was 3/15/2024. The choice of bendamustine lymphodepletion was not driven by specific patient characteristics but rather it has been our predominant lymphodepletion strategy due to our extensive experience [6, 9] coupled with fludarabine shortage [10]. Outcome and laboratory data were obtained from electronic medical records. Response was assessed according to Lugano criteria and toxicities using ASTCT criteria and CTCAE v5.0. This retrospective study was approved by the Institutional Review Board.

**Table 1:** Patients’ characteristics

Characteristic		Total 31 (100%)
**Age at infusion**	≤ 65 years	16 (51.6%)
	> 65 years	15 (48.4%)
	Median [IQR]	63 [61–76]
**Diagnosis**	DLBCL NOS	18 (58.1%)
	HGBCL with MYC and BCL2 and/or BCL6 rearrangements	2 (6.5%)
	PMBCL	1 (3.2%)
	DLBCL transformed from indolent lymphomas	8 (25.8%)
	T cell histiocyte rich lymphoma	1 (3.2%)
	FL Grade 3b	1 (3.2%)
**Sex**	Female	14 (45.2%)
	Male	17 (54.8%)
**# of previous therapies**	1	12 (38.7%)
	2	6 (19.4%)
	≥ 3	13 (41.9%)
**Previous ASCT**	No	28 (90.3%)
	Yes	3 (9.7%)
**Bridging therapy**	No	7 (22.6%)
	Yes	24 (77.4%)
**ECOG grade**	≤ 1	28 (90.3%)
	> 1	3 (9.7%)
**Status at last disease evaluation**	CR	8 (25.8%)
	PR	12 (38.7%)
	SD	2 (6.5%)
	PD	9 (29.0%)
Bulky disease* at last evaluation	No	29 (93.5%)
	Yes	2 (6.5%)
**LDH levels at liso-cel infusion**	Normal	20 (64.5%)
	Elevated	11 (35.5%)
**Platelet count at liso-cel infusion**	≥ 50 × 10^9^/L	30 (96.8%)
	< 50 × 10^9^/L	1 (3.2%)
**TRANSCEND-001-NHL inclusion criteria** (***n*** = **19)**	Yes	16 (84.2%)
	No	3 (15.8%)
**TRANSFORM inclusion criteria** (***n*** = **12)**	Yes	8 (66.7%)
	No	4 (33.3%)

*Abbreviations*: ASCT: autologous stem cell transplant; Bulky disease: largest diameter of disease localization > 10 cm; CR: Complete response; DLBCL: diffuse large B cell lymphoma; FL: follicular lymphoma; ECOG PS: Performance status according to Eastern Cooperative Oncology Group; HGBCL: High grade B cell lymphoma; IQR: interquartile range; LDH: Lactate dehydrogenase; Liso-cel: lisocabtagene maraleucel; n: number; NOS: not otherwise specified; PD: progressive disease: PMBCL: primary mediastinal B cell lymphoma; PR: Partial response; SD: Stable disease

**Fig. 1:**
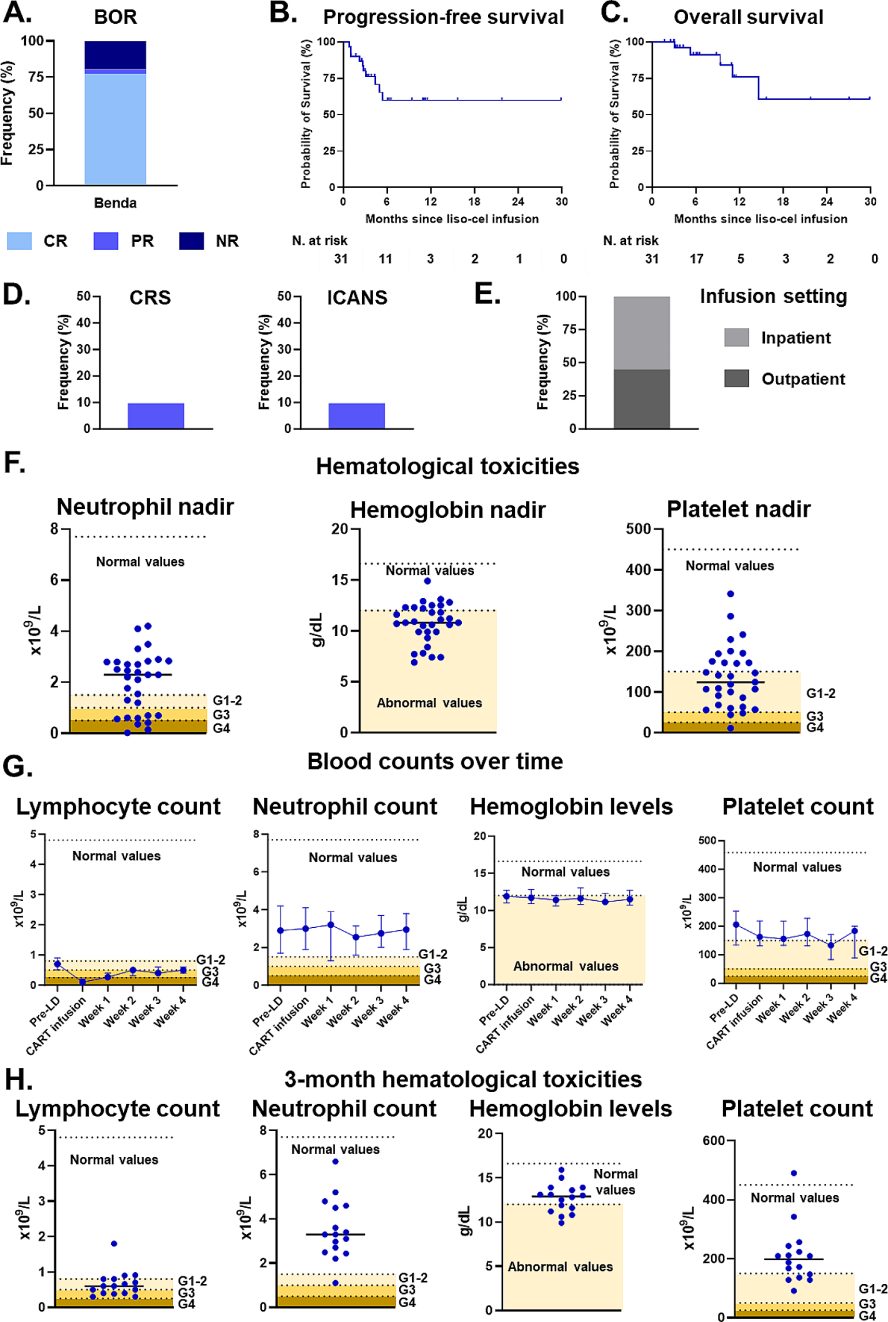
**Efficacy and safety of bendamustine lymphodepletion before lisocabtagene maraleucel. A.** Best response after liso-cel. Light blue represents complete response, blue represents partial response, and dark blue represents no response. **B.** Progression-free survival after liso-cel. **C.** Overall survival after liso-cel. **D**. CRS and ICANS of any grade incidence. **E**. Infusion setting of liso-cel infusion. Light gray represents the inpatient setting and dark gray represents outpatient setting. **F**. Hematologic toxicities within 30 days after liso-cel infusion. Dot plots highlight individual nadir values of neutrophils, hemoglobin and platelets levels. Shadows of yellow background highlight the range of specific abnormal levels. **G**. Blood counts over time during the 4 weeks after liso-cel infusion. Values are expressed as median and 95% confidence interval error bars. Shadows of yellow background highlight the range of specific abnormal levels. **H**. Blood counts at 3 months after liso-cel infusion. Dot plots highlight individual nadir values of lymphocytes, neutrophils, hemoglobin, and platelets levels. Shadows of yellow background highlight the range of specific abnormal levels *Abbreviations*: CR: complete response; CRS: cytokine release syndrome; ICANS: immune effector cell-associated neurotoxicity syndrome; N: number; NR: not response; PR: partial response

Seven out of 31 patients (22.6%) would not have met the inclusion criteria for the liso-cel clinical trials [3, 5], due to previous CART19 treatment (*n* = 2), older age (*n* = 4), and elevated bilirubin (*n* = 1). Conversely, histologies slightly differed from registration studies. The median time from apheresis to infusion was 43 days (range: 32–150). The best response rate was complete response for 77.4% of patients, partial response for 3.2%, and no response for 19.4% (Fig. 1A). With a median follow-up of 6.3 months, the 6-month progression-free survival (PFS) and overall survival were 59.9% (Fig. 1B) and 91.1% (Fig. 1C), respectively. The incidence of cytokine-release syndrome (CRS) was 9.7% (all grade 1) and immune effector cell associated neurotoxicity syndrome (ICANS) was 9.7% (two cases of grade 1 and one of grade 2). There were no cases of grade ≥ 3 CRS or ICANS (Fig. 1D). No infections were observed during the first 30 days after liso-cel. Fourteen patients (45.2%) received liso-cel infusion in the outpatient setting (Fig. 1E). The median hospitalization time was 8 days. The median absolute lymphocyte count (ALC) at the time of liso-cel infusion was 0.10 × 10^9^/L (IQR 0.09–0.20). The median absolute neutrophil count (ANC) nadir was 2.30 × 10^9^/L (IQR 0.70–2.80), with 29.0% of patients with grade ≥ 3 neutropenia. The median hemoglobin nadir was 10.8 g/dL (IQR 9.9–12.3). The median platelet nadir was 124 × 10^9^/L (IQR 68–175), with 9.7% of patients with grade ≥ 3 thrombocytopenia (Fig. 1F). Neutrophils, hemoglobin, and platelet counts were generally stable during the four weeks after liso-cel infusion while lymphocytes progressively recovered over time (Fig. 1G). At 3 months, among the 16 patients with ongoing responses, we observed median ALC of 0.60 × 10^9^/L (IQR 0.40–0.80), median ANC 3.30 × 10^9^/L (IQR 2.54–4.56), median hemoglobin 12.9 g/dL (IQR 11.3–13.8), and median platelet count 198 × 10^9^/L (IQR 139–238) (Fig. 1H).

In conclusion, we observed that bendamustine lymphodepletion was overall effective and associated with manageable toxicities. The median PFS in our analysis was not reached, as compared to that of the phase 2 TRANSCEND-NHL-001 that was 6.8 months [3, 4] and of the phase 3 TRANSFORM trials, i.e., 10.1 months [5]; however, the median follow-up for the present study is significantly shorter and patients received liso-cel both in 2nd and >3rd line. In our cohort, incidence of CRS and ICANS of any grade were 9.7% and 9.7%, respectively, while in the TRANSCEND-NHL-001 they were 42% and 30%, and in the TRANSFORM 49% and 11%, respectively [3, 5]. Although different CRS criteria were used, the overall incidence and severe cases can be compared as shown by Pennisi et al. [11]. No patients received tocilizumab. ICANS was effectively treated with steroids. Neutropenia of grade ≥ 3 was 60% in TRANSCEND-NHL-001 [3] and 82% in TRANSFORM [5] versus 29% in the present study. Indeed, no infection-related events were observed in our cohort, while 12% and 15% of patients in the TRANSCEND-NHL-001 and TRANSFORM, respectively, developed severe infections [3–5]. These data are in line with previous publications demonstrating that bendamustine lymphodepletion is associated with reduced incidences of CRS, ICANS, hematological toxicities, and infections compared with Flu/Cy [6, 8, 9, 12].

## Data Availability

All requests for raw and analyzed data and materials will be promptly reviewed by the University of Pennsylvania to determine if they are subject to intellectual property or confidentiality obligations. Patient-related data may be subject to patient confidentiality. Any data and materials that can be shared will be released via a material transfer agreement. Other data generated from this study are available from the corresponding author upon reasonable request.

## Abbreviations

ALC: Absolute lymphocyte count
ANC: Absolute neutrophil count
CART19: Anti-CD19 chimeric antigen receptor T cell therapy
CRS: Cytokine-release syndrome
Flu/Cy: Fludarabine and cyclophosphamide lymphodepletion
ICANS: Immune effector cell associated neurotoxicity syndrome
IQR: Interquartile range
LBCL: Large B cell lymphoma
Liso-cel: Lisocabtagene maraleucel
PFS: Progression-free survival
